# Implications of climate-related disasters on refugees’ health: A case study of resettled Syrian and Iraqi refugees in San Diego, California

**DOI:** 10.21203/rs.3.rs-3392999/v1

**Published:** 2023-10-03

**Authors:** Behnan Albahsahli, Anna Dimitrova, Nadine Kadri, Tarik Benmarhnia, Tala Al Rousan

**Affiliations:** Herbert Wertheim School of Public Health, University of California, San Diego, USA; Scripps Institution of Oceanography, University of California, San Diego, USA; Western University of Health Sciences, Pomona, California, USA; Scripps Institution of Oceanography, University of California, San Diego, USA; Herbert Wertheim School of Public Health, University of California, San Diego, USA

## Abstract

**Background:**

Climate change disproportionately harms people of color and low-income communities. Despite their unprecedented numbers, being constantly on the move, and suffering extreme social vulnerability, almost nothing is known about the impact of climate change on the health of refugees. This study uses state-of-the-art mixed methods to examine the differential susceptibility of climate-sensitive exposures and environmental exposures among refugees and their links to perceived health after resettlement.

**Methods:**

Arabic-speaking refugees (N=67) from Iraq and Syria previously diagnosed with hypertension who resettled in California were recruited from a community center. Semi-structured interviews were conducted to explore participant’s understanding of the impact of climate on health. Survey data were collected to inquire regarding participant’s refugee journeys prior to resettlement in the US. Survey data on climate-related disasters was retrospectively geo-referenced through the Emergency Events Database (EM-DAT). Qualitative data was analyzed using inductive thematic analysis.

**Results:**

All participants stayed in at least one temporary resettlement country during their migration journey and 12% has stayed in refugee camps. The most popular resettlement sites were Turkey (most disaster-prone in the region due to frequent floods and earthquakes) and Jordan (one of the most extreme water-scarce globally). Participants reported harsh weather conditions during their migratory journeys including extreme cold in Turkey and extreme heat in Jordan. Many participants noted their exposure to dust throughout their travels, and an inability to deal with harsh weather conditions due to financial insecurity. Participants did not link their diagnosis of hypertension to their experience of extreme weather and would only link it to their exposure to stress from war. Participants did note poorer mental health due to poor weather conditions and a challenge adjusting to the climate conditions in different countries. Few participants reported residing in a refugee camp and described it as ill-equipped for the challenges of climate hazards.

**Conclusion:**

This study reveals the links between structural drivers of climate change and health inequities for refugee populations. Refugees are highly vulnerable to climate-sensitive exposures but remain not fully aware of the potential links between these exposures and health. Learnings from this study will inform clinical and public health interventions, and policies to close the climate gap without leaving this vulnerable population behind.

The climate crisis poses multiple threats to human health^1^, with those who have contributed the least to it generally suffering the gravest consequences^2^. Refugees are among the population groups least equipped to protect themselves from environmental hazards due to a combination of factors and geographical trajectories. These can include insecure living conditions, such as living in overcrowded refugee camps, a lack of economic resources, the experience of conflict and persecution in their place of origin, and often extremely perilous journeys. Previous research shows that refugees have a greater prevalence of poor physical^3^ and mental health^4^ as compared to the general population, including chronic conditions such as hypertension^3^, which are shown to be aggravated by exposure to extreme climate events^5^. Yet little is known about the implications of the climate crisis on refugees’ health, mainly due to the difficulty of studying displaced populations as well as their previous exposures to climate-related disasters. Understanding and documenting such refugees’ cumulative exposure to various environmental exposures can be particularly helpful to better understand differential susceptibility regarding the role of climate-sensitive exposures and ambient environmental exposures once resettled in the United States (US) or elsewhere.

In this study, we demonstrate how some of these gaps in the literature can be addressed through a mixed-methods approach applied to a small sample of Syrian and Iraqi refugees. Semi-structured interviews were conducted with 67 adult refugees residing in San Diego, California – a major resettlement city for refugees from the Middle East and North Africa (MENA) in the US. The sociodemographic characteristics of interviewed refugees are presented in Table S1. These data were analyzed through inductive thematic analysis alongside geo-referenced data on climate-related disasters retrieved from the Emergency Events Database (EM-DAT) (see supplementary information). Participants were asked about their refugee journeys prior to resettlement in the United States, which span from 1992 to 2018, including the experience of climate-related hazards and perceived health implications.

The MENA region is important to study since it has been badly affected by conflicts and is a major contributor to the global refugee crisis, with more than 12 million people currently displaced in the region^6^. At the same time, this region is warming significantly faster than other regions and incurs some of the highest rates of human losses from the climate crisis due to its poor adaptive capacity^7^.

Most of the surveyed individuals stayed in at least at one temporary resettlement site in the MENA region during their migratory journey, primarily in Turkey and Jordan (figures 1A,1G). Besides being the top resettlement country per capita, Turkey is among the most disaster-prone countries in the region (figure 1C,1D). The frequent and catastrophic floods there are of great concern for refugees because of the potential for further displacement, the difficulty providing humanitarian assistance, and the rapid spread of communicable diseases.

Surveyed participants reported experiencing harsh weather conditions, including extreme heat in in Jordan and extremely cold temperatures in Turkey. Access to heating during cold winters was noted as a problem due to a lack of financial resources. Almost all participants mentioned being exposed to dust throughout their resettlement journey. Such dust storms are becoming more common in the Middle East due to the climate crisis^8^. Both dust and extreme ambient temperatures can aggravate hypertension, which was a chronic condition among all survey participants. While some related their hypertension to the experience of war and violence, no direct links were made between the experience of extreme weather during the migratory journey and poor physical health. However, some refugees reported poor mental health due to poor environmental conditions. Overall, participants reported difficulties adjusting to a climate that was different from their home countries.

A few survey participants reported staying in a refugee camp in Jordan (figure 1F) and generally spoke negatively of their experiences there, describing conditions in the camp as ill-equipped for the desert climate in the region. The presence of dust, lack of clean water, and poor protection from the outside environment in the temporary tent structures were emphasized. Several participants reported suffering from asthma during their stay due to poor air quality and other illnesses related to contact with contaminated water. Although refugee camps are intended to be temporary resettlement sites, it is not uncommon for refugees to spend decades there^9^ in extremely precarious living conditions^10^.

The impact of environmental disasters on refugees’ health is an immensely understudied field of research. Applying a mixed-methods approach, we propose a framework to study such links and highlight specific health risks faced by refugees in the MENA region where violent conflict has displaced millions of people against the backdrop of an intensifying climate crisis. As climate change is expected to displace an ever-growing number of people in the next decades, there is an urgent need to develop actionable interventions to improve the quality of life and health of this highly vulnerable group.

## Figures and Tables

**Figure 1:**
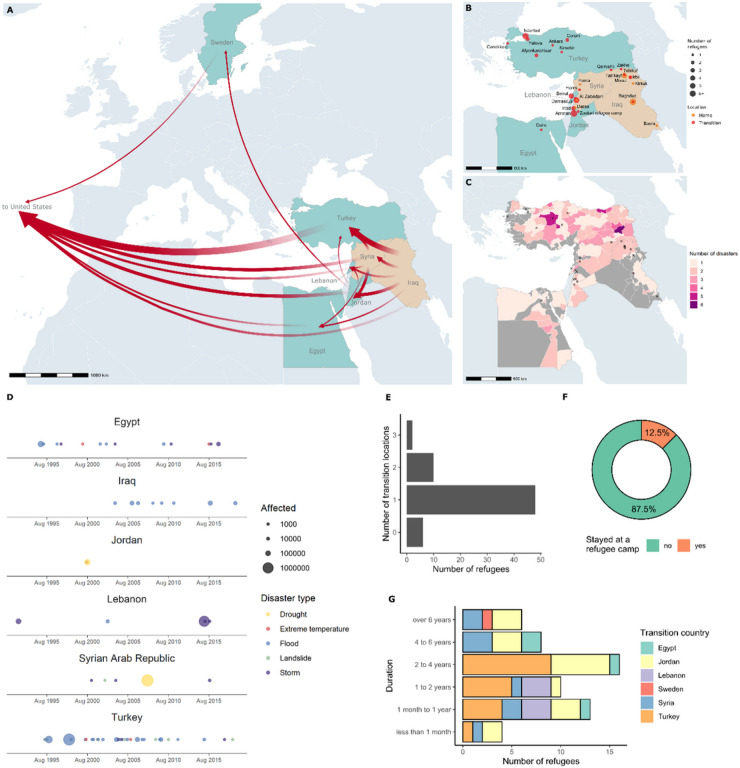
Migration routes from Syria and Iraq to the United States reported by the interviewed refugees (A), home and transition locations prior to resettlement in the United States (B), number of climate-related disasters per subnational administrative unit in transition countries (C), occurrence and impacts of climate-related disasters per transition country (D), and descriptive statistics for the sample of interviewed refugees (E-G). Data on disasters are based on own calculations of climate-related disasters (storms, floods, landslides, extreme temperatures, and droughts) from the Emergency Events Database (EM-DAT) by the Center for Research on the Epidemiology of Disasters (CRED) at the School of Public Health of the Universite Catholique de Louvain in Belgium.
